# Early versus delayed initiation of adjuvant treatment for pancreatic cancer

**DOI:** 10.1371/journal.pone.0173960

**Published:** 2017-03-16

**Authors:** Hyoung Woo Kim, Jong-Chan Lee, Jongchan Lee, Jin Won Kim, Jaihwan Kim, Jin-Hyeok Hwang

**Affiliations:** 1 Department of Internal Medicine, Seoul National University College of Medicine, Seoul National University Bundang Hospital, Seongnam, Korea; 2 Department of Internal Medicine, Chungbuk National University Hospital, Chungbuk National University College of Medicine, Cheongju, Korea; University of Nebraska Medical Center, UNITED STATES

## Abstract

**Background:**

Pancreatic ductal adenocarcinoma (PDAC) is a highly aggressive tumor showing a tendency for early recurrence, even after curative resection. Although adjuvant treatment improves survival, it is unclear whether early adjuvant treatment initiation yields better outcomes in patients with PDAC.

**Methods:**

We retrospectively enrolled 113 patients who underwent chemotherapy or chemoradiotherapy after curative resection of PDAC: Fifty-six and 57 patients were in the early and delayed groups, respectively based on the median time of treatment initiation (35 days [range, 20–83 days]).

**Results:**

Patient baseline characteristics were comparable in both groups, except for grade III or IV postoperative complications (5.4% in the early group vs. 22.8% in the delayed group). With a median 20.3-month follow-up, the overall survival (OS) and disease-free survival (DFS) times were 29.5 and 14.7 months, respectively. The early group had significantly prolonged OS (39.1 vs. 21.1 months, p = 0.018) and DFS (18.8 vs. 10.0 months, p = 0.034), compared to the delayed group. Among 71 patients who completed planned adjuvant treatment, patients in the early group tended to have longer, though not statistically significant, OS and DFS times than those in the delayed group. In 67 patients without postoperative complications, patients in the early group had longer OS (42.8 vs. 20.5 months, p = 0.002) and DFS (19.6 vs. 9.1 months, p = 0.005) than those in the delayed group. By multivariate analysis, incompletion of treatment (hazard ratio [HR]: 4.039, 95% confidence interval [CI]: 2.334–6.992), delayed treatment initiation (HR: 1.822, 95% CI: 1.081–3.070), and positive angiolymphatic invasion (HR: 2.116, 95% CI: 1.160–3.862) were significantly associated with shorter OS.

**Conclusions:**

Adjuvant treatment should be delivered earlier and completed for better outcomes in resected PDAC patients, especially without postoperative complications.

## Introduction

Pancreatic ductal adenocarcinoma (PDAC) is one of the most fatal solid tumors, with a 5-year survival rate of less than 6% [1, 2]. Although most patients with locally advanced or metastatic PDAC die within 5 years, a few patients with localized PDAC can achieve long-term survival, if complete resection and adjuvant treatment can be administered [3, 4]. Adjuvant treatment is mandatory because it more than doubles the 5-year survival rate, from approximately 10% with surgery alone to 25% with adjuvant chemotherapy following surgery [5–10]. However, the appropriate time to initiate adjuvant treatment, considering patient’ safety, compliance and effectiveness, has not been well described in PDAC patients to date.

Recent retrospective data from the ESPAC-3 study found that completion of six cycles of chemotherapy was independently associated with longer survival [11]. Furthermore, early chemotherapy initiation would be more harmful than delayed initiation of chemotherapy in patients with incomplete scheduled chemotherapy. Therefore, they suggested that chemotherapy should be considered after adequate recovery. However, theoretically, micrometastatic deposits are eliminated more effectively with earlier chemotherapy administration, thereby reducing early recurrence [12]. Moreover, there is evidence that early adjuvant treatment initiation leads to superior survival rates for patients with breast [13, 14], colorectal [15–17], and pancreatic cancer [12, 18]. Thus, the National Comprehensive Cancer Network (NCCN) guidelines state that chemotherapy should be initiated for all patients who underwent curative resection, but they do not specify the optimal start time for chemotherapy or whether it should be delayed until full recovery [19]. In clinical trials and practice, most patients received adjuvant treatment within 4–8 weeks unless postoperative complications occurred, and there is an apparent disagreement in the appropriate time to receive adjuvant treatment between guidelines (within 3 months) and clinical trials (within 4–8 weeks) [5–10].

The current study evaluated whether early initiation of adjuvant treatment could yield better outcomes than delayed initiation in patients with resected PDAC and to determine the importance of scheduled treatment completion.

## Patients and methods

### Patients

**Fig 1:**
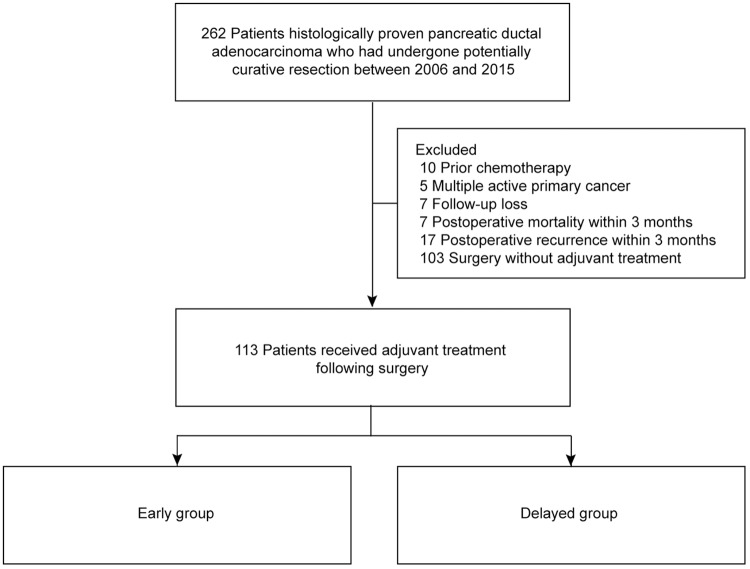
Patient flow diagram. Enrolled patients were divided into early and delayed groups, according to the median time of adjuvant treatment initiation.

Data were obtained from 262 consecutive patients with PDAC who underwent curative resection at Seoul National University Bundang Hospital (Seongnam, Korea) between January 2006 and May 2015. Since 2010, adjuvant treatment has been done universally [5–8]. After excluding 149 patients for prior chemotherapy, multiple active primary cancer, follow-up loss, mortality or recurrence within 3 months after surgical resection, and surgery without adjuvant treatment, 113 patients who underwent curative surgical resection followed by adjuvant treatment were enrolled retrospectively. According to the median time of adjuvant treatment initiation (TT), enrolled patients were divided into early and delayed groups (Fig 1). The study protocol was reviewed and approved by the Institutional Review Board (IRB) of Seoul National University Bundang Hospital (IRB no.: B-1605-349-101). Our institutional review board waived the need for written informed consent from the participants.

### Outcomes

The outcomes for this study were overall survival (OS) and disease-free survival (DFS) in both groups. OS was defined as the time from the date of surgery to the date of death. DFS was defined as the time from the date of surgery to the date of the first documentation of recurrence or death, if either event occurred before documented radiological or histological recurrence. Patients alive without recurrence were censored on the date of the last follow-up.

### Data collection, postoperative management, and surveillance

Patient characteristics included age, sex, comorbidities, and preoperative laboratory exam results. Additionally, surgical data, pathologic data, strategy and regimens of adjuvant treatment, TT, postoperative complications, and postoperative recurrence or mortality were collected. Comorbidities were assessed by the Charlson age-adjusted comorbidity index (CACI) [20, 21]. All patients underwent contrast-enhanced abdominal-pelvic computed tomography (CT) to evaluate postoperative complications within 4 weeks. Postoperative complications were defined as events that required additional treatment within 3 months of surgery, based on the Clavien-Dindo classification [22, 23]. Grade III–IV complications were defined as major if they required surgical, endoscopic, or radiological intervention, regardless of organ dysfunction. After wound healing, patients received adjuvant chemotherapy and/or concurrent chemoradiotherapy within 3 months of surgery. Gemcitabine alone, 5-fluorouracil (FU) plus leucovorin, or concurrent chemoradiotherapy (gemcitabine, 5-FU, or capecitabine-based chemoradiotherapy) was used as adjuvant treatment for most patients [5, 7–10, 19]. Most patients underwent laboratory tests and contrast-enhanced abdominal-pelvic CT every 8–12 weeks for the first 2 years. If patients had no apparent recurrence during that time, they were then followed at 6-month intervals [19]. If documented radiological or histological recurrence occurred, patients received palliative chemotherapy or the best supportive care. Various regimens, such as FOLFIRINOX, FOLFOX, XELOX, gemcitabine alone, gemcitabine plus erlotinib, gemcitabine plus cisplatin, and capecitabine, were used as first-line palliative chemotherapeutic regimens.

### Statistical analysis

Differences in continuous and non-continuous variables between the early and delayed groups were compared using Student’s t-test and χ^2^ tests, respectively. Kaplan-Meier analysis was used to generate survival curves and calculate median survival times, which were compared using the log-rank test. The risk of death or recurrence was compared using the log-rank test and the Cox proportional hazards regression model. Risks are expressed as hazard ratios (HRs). Among the clinical variables included in univariate analyses, those with a two-sided P-value <0.05 were chosen for multivariate analyses with stepwise selection. A two-sided P-value <0.05 was considered statistically significant. All statistical analyses were performed using STATA 14.0 software (STATA Corp., College Station, TX).

## Results

### Patient characteristics

Among 262 who had undergone potentially curative resection, 46 patients were excluded due to prior chemotherapy, synchronous malignancies, follow-up loss and early mortality or recurrence (<3 months), and 103 patients who did not receive adequate adjuvant treatment due to delayed postoperative recovery (poor performance) or patients’ decision were excluded as well (Fig 1). For the 113 patients (chemotherapy alone, 70 [61.9%]; concurrent chemoradiotherapy, 43 [38.1%]), the median TT was 35 days (range, 20–83 days). In total, 71 patients (62.8%) completed six cycles of adjuvant treatment. Fifty-six patients were in the early group (TT <35 days), and 57 patients were in the delayed group (TT ≥35 days) (Fig 1 and S1 Fig). The median TTs were 28 days in the early group and 42 days in the delayed group. Most patients (104/113, 92.0%) received adjuvant treatment within 8 weeks.

Patient baseline characteristics were comparable in both groups in terms of age, sex, comorbidity index, surgical data, pathologic features, and adjuvant treatment, with the exception of grade III–IV postoperative complications (5.4% in the early group vs. 22.8% in the delayed group) (Table 1).

**Table 1:** Patient baseline characteristics.

Characteristic, no. (%)	Timing of the adjuvant treatment		
	Early group	Delayed group	*P* value
No. of patients	56	57	
Age, median (range), years	60 (38–80)	66 (43–77)	0.148
Sex			0.746
Female	19 (33.9)	21 (36.8)	
Male	37 (66.1)	36 (63.2)	
CACI			0.662
<6	54 (96.4)	54 (94.7)	
≥6	2 (3.6)	3 (5.3)	
Preoperative CA19-9			0.774
≤100 U/mL	27 (47.3)	29 (50.0)	
>100 U/mL	29 (52.7)	28 (50.0)	
Time of surgery			0.112
2006–2009 year	8 (14.3)	15 (26.3)	
2010–2015 year	48 (85.7)	42 (73.7)	
Surgical procedure			0.333
Laparoscopic resection	14 (25.0)	10 (17.5)	
Open resection	42 (75.0)	47 (82.5)	
Type of resection			0.383
DP	20 (35.7)	16 (28.1)	
PD	36 (64.3)	41 (71.9)	
Operating time			0.844
≤500 minutes	46 (82.1)	46 (80.7)	
>500 minutes	10 (17.9)	11 (19.3)	
Intraoperative transfusion			0.346
No	48 (85.7)	45 (78.9)	
Yes	8 (14.3)	12 (21.1)	
Longest diameter of primary tumor			0.194
≤2 cm	12 (21.4)	7 (12.3)	
>2 cm	44 (78.6)	50 (87.7)	
Nodal status			0.153
Negative	25 (44.6)	18 (31.6)	
Positive	31 (55.4)	39 (68.4)	
Resection margin status			0.653
R0	47 (83.9)	46 (80.7)	
R1	9 (16.1)	11 (19.3)	
Differentiation			0.999
Well	5 (8.9)	5 (8.8)	
Moderate	44 (78.6)	45 (78.9)	
Poor	7 (12.5)	7 (12.3)	
Angiolymphatic invasion			0.159
Negative	31 (55.4)	24 (42.1)	
Positive	25 (44.6)	33 (57.9)	
Venous invasion			0.911
Negative	33 (58.9)	33 (57.9)	
Positive	23 (41.1)	24 (42.1)	
Perineural invasion			0.592
Negative	6 (10.7)	8 (14.0)	
Positive	50 (89.3)	49 (86.0)	
Postoperative complications			0.024
No	38 (67.9)	29 (50.9)	
Grade I/II	15 (26.8)	15 (26.3)	
Grade III/IV	3 (5.4)	13 (22.8)	
Adjuvant treatment			0.153
Chemotherapy alone	31 (55.4)	39 (68.4)	
Concurrent chemoradiotherapy	25 (44.6)	18 (31.6)	
Completed 6 cycles of treatment			0.273
Yes	38 (67.9)	33 (57.9)	
No	18 (32.1)	24 (42.1)	
Median follow-up, months (range)	23.5 (7.0–119.7)	16.5 (5.8–114.4)	0.230

CACI, Charlson age-adjusted comorbidity index; CA19-9, carbohydrate antigen 19–9; DP, distal pancreatectomy; PD, pancreaticoduodenectomy; R0, macroscopically and microscopically negative resection margin; R1, microscopically positive resection margin.

### OS and DFS in the early and delayed groups

**Fig 2:**
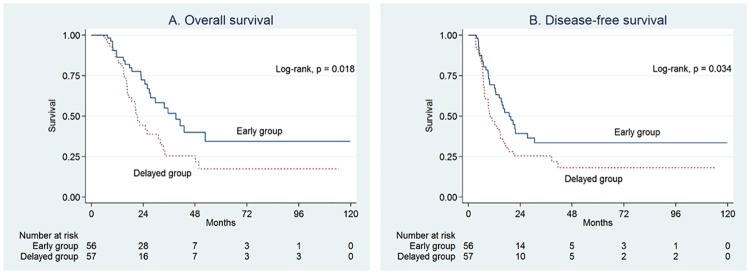
Kaplan-Meier curves of overall survival (A) and disease-free survival (B). The early group consisted of patients who received early initiation of adjuvant treatment (<35 days), and the delayed group consisted of patients who received delayed initiation of adjuvant treatment (≥35 days).

The median OS and DFS times for the overall study population were 29.5 and 14.7 months, respectively, during the median 20.3-month follow-up. The early group had a significantly longer OS than the delayed group (39.1 vs. 21.1 months, P = 0.018), and a longer DFS than the delayed group (18.8 vs. 10.0 months, P = 0.034) (Fig 2). Additionally, of the 104 patients receiving adjuvant treatment within 8 weeks, patients who received earlier treatment (TT <33 days) had significantly longer OS and DFS than those who did not (TT ≥33 days) (P = 0.043 and = 0.005, respectively) (S2 Fig). By multivariate Cox regression analysis, lack of adjuvant treatment completion, delayed adjuvant treatment initiation, and positive angiolymphatic invasion were independent prognostic factors for OS and DFS (Table 2 and S1 Table). There were no significant differences in the reasons for discontinuing adjuvant treatment between early and delayed groups (S2 Table).

**Table 2:** Risk factors for overall survival.

Risk factor	No.	Univariate analysis		Multivariate analysis	
		HR (95%CI)	*P* value	HR (95%CI)	*P* value
Timing of the adjuvant treatment					
Early initiation	56				
Delayed initiation	57	1.880 (1.107–3.191)	0.019	1.822 (1.081–3.070)	0.024
Age, years					
≤65	65				
>65	48	0.698 (0.398–1.224)	0.698		
Sex					
Female	40				
Male	73	1.544 (0.861–2.770)	0.145		
CACI					
<6	108				
≥6	5	1.541 (0.372–6.380)	0.551		
Preoperative CA19-9					
≤100 U/mL	56				
>100 U/mL	57	1.315 (0.776–2.229)	0.309		
Time of surgery					
2006–2009 year	23				
2010–2015 year	90	1.037 (0.588–1.828)	0.901		
Surgical procedure					
Laparoscopic resection	24				
Open resection	89	2.342 (0.934–5.872)	0.070		
Type of resection					
DP	36				
PD	77	1.058 (0.611–1.831)	0.841		
Operating time					
≤500 minutes	92				
>500 minutes	21	2.472 (1.343–4.553)	0.004	1.357 (0.696–2.645)	0.371
Intraoperative transfusion					
No	93				
Yes	20	1.577 (0.852–2.917)	0.147		
Longest diameter of primary tumor					
≤2 cm	19				
>2 cm	94	1.849 (0.837–4.083)	0.128		
Nodal status					
Negative	43				
Positive	70	2.062 (1.166–3.647)	0.013	1.675 (0.934–3.003)	0.083
Resection margin status					
R0	93				
R1	20	1.168 (0.589–2.315)	0.657		
Differentiation					
Well or moderate	99				
Poor	14	0.769 (0.329–1.795)	0.543		
Angiolymphatic invasion					
Negative	55				
Positive	58	1.866 (1.103–3.157)	0.020	2.116 (1.160–3.862)	0.015
Venous invasion					
Negative	66				
Positive	47	1.837 (1.085–3.109)	0.024	0.961 (0.519–1.782)	0.961
Perineural invasion					
Negative	14				
Positive	99	2.155 (0.858–5.409)	0.102		
Postoperative complications					
No	67				
Grade I/II	30	1.107 (0.593–2.069)	0.749		
Grade III/IV	16	1.537 (0.734–3.219)	0.254		
Completed 6 cycles of treatment					
Yes	71				
No	42	4.040 (2.375–6.873)	<0.001	4.039 (2.334–6.992)	<0.001

HR, hazard ratio; CI, confidence interval; CACI, Charlson age-adjusted comorbidity index; CA19-9, carbohydrate antigen 19–9; DP, distal pancreatectomy; PD, pancreaticoduodenectomy; R0, macroscopically and microscopically negative resection margin; R1, microscopically positive resection margin.

**Fig 3:**
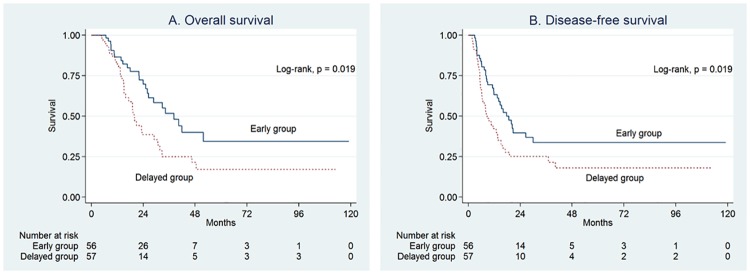
Kaplan-Meier curves of overall survival (A) and disease-free survival (B) based on the starting point of the survival interval (date of adjuvant treatment).

Survival analyses were conducted by analyzing the survival interval both from the date of surgery and from the date of adjuvant treatment initiation; to avoid lead-time bias. As expected, both OS and DFS were longer in the early group than in the delayed group (Fig 3).

### OS and DFS in patients according to planned treatment completion

**Fig 4:**
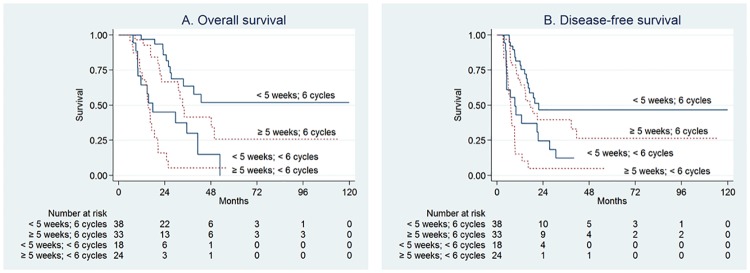
Kaplan-Meier curves of overall survival (A) and disease-free survival (B) in patients who received all six planned cycles of treatment and those who did not. There was no difference in overall or disease-free survival for patients who received all six planned cycles of therapy (P = 0.129 and = 0.195, respectively) and those who did not (P = 0.206 and = 0.133, respectively).

OS and DFS times were compared between patients who received all six cycles of planned treatment and those who did not (Fig 4). The former group had a significantly longer OS (42.8 vs. 15.5 months, P <0.001), and DFS (19.6 vs. 7.1 months, P <0.001) than the latter group. In the 71 patients who completed planned adjuvant treatment, TT did not influence OS and DFS significantly, although the patients who received early treatment tended to have a longer OS and DFS than those who did not (Fig 4).

**Fig 5:**
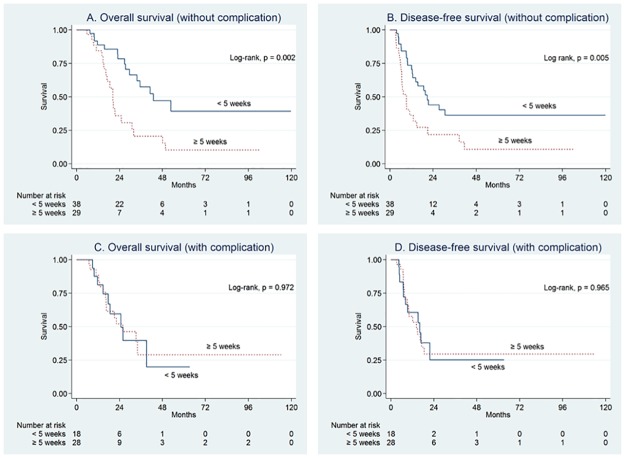
The effect of time to adjuvant treatment initiation on overall survival (A, C) and disease-free survival (B, D) in patients without or with postoperative complications, respectively.

Given the observed correlation between postoperative complications and delayed adjuvant treatment initiation, survival analysis was conducted for complication-dichotomized groups. As shown in Fig 5, early initiation compared with delayed initiation of adjuvant treatment was significantly associated with a superior survival rate for patients without postoperative complications, while no significant association was noted for those with complications. Additionally, there was no survival difference between patients who received chemotherapy and those who received chemoradiotherapy as adjuvant treatment (S3 Fig).

## Discussion

Adjuvant treatment is mandatory in patients who undergo curative resection of PDAC. However, previously published data do not specifically indicate how late adjuvant treatment could be delayed without a decrease in efficacy or how early it could be delivered without a decrease in compliance. We evaluated whether or not patients with resected PDAC who received early adjuvant treatment could expect better clinical outcomes than patients who received delayed treatment. For patients who began adjuvant treatment earlier (within 5 weeks after surgery), OS and DFS were increased without a compliance decrease (completion rate: 67.9% in the early group vs. 57.9% in the delayed group). Moreover, the patients who received early treatment and completed all six cycles of treatment tended to have a longer OS and DFS than those who did not, although it did not reach statistical significance.

Although surgery is the gold standard for management of localized PDAC, some experimental evidence suggests that surgery could have some disadvantages in terms of tumor biology, such as stimulation of growth factor release and suppression of cytotoxic T-cell and natural killer cell activation [24–28]. This may lead to angiogenesis, accelerated micrometastasis, or activation of occult metastatic tumor cells [29]. Thus, the rationale for adjuvant treatment includes the prevention of micrometastasis, especially for pancreatic cancer, which is characterized by early micrometastasis and aggressiveness. A recent study showed that the numbers of primary and metastatic tumor cells were inversely proportional to the time between surgery and the initiation of adjuvant treatment [12]. Furthermore, the authors showed that earlier initiation of adjuvant treatment was associated with proportionally longer survival due to a reduction the number of tumor cells [12]. As long as patients are fully recovered, theoretically, early initiation of adjuvant treatment should provide better results. From another standpoint, we were concerned that the early initiation of adjuvant treatment is not always beneficial because pancreaticoduodenectomy is a highly morbid surgery with a high complication rate, and patients require a longer time to recover. In the current study, most patients (92.0%) received adjuvant treatment within 8 weeks after full recovery, and 62.8% patients (67.9% in the early group vs. 57.9% in the delayed group) completed the planned six cycles of adjuvant treatment although patients with postoperative complications tended to receive adjuvant treatment later. This result shows that adjuvant treatment could be delivered without a decrease in compliance despite a short TT of less than 5 weeks.

According to previous data from the ESPAC-3 study, most patients with resected PDAC might not fully recover from the pancreatic cancer surgery within 8 weeks, and those patients were predicted to be at a disadvantage because they could not complete the planned six cycles of adjuvant treatment [11]. However, the ‘8 weeks’ is quite different from our median TT ‘5 weeks’ and most patients (92.0%) received adjuvant treatment within 8 weeks in the current study. However, earlier start of adjuvant treatment in our study could lower completion rate compared with ESPAC-3 study (67.9% vs. 73% in the early group; 57.9% vs. 67% in the delayed group). Additionally, the current study did further evaluate outcomes according to the postoperative complications. In patients without postoperative complications, the earlier initiation could provide better OS and DFS. Based on our observations and previous *in vivo* & *in vitro* data [12], we believe that earlier adjuvant treatment initiation would provide better outcomes, as long as patients are fully recovered.

There were some limitations to the current study. First, the current study was a retrospective study that examined a relatively small number of patients. However, both groups were well balanced and had comparable baseline characteristics. A prospective study would be difficult to perform because of ethical considerations. Secondly, approximately one-third of all patients received adjuvant chemoradiotherapy.

## Conclusions

We demonstrated that earlier adjuvant treatment initiation and treatment completion were associated with better survival rates in patients with resected PDAC, although completion of the scheduled treatment was the strongest prognostic factor. Furthermore, earlier adjuvant treatment initiation should be considered, as long as patients have good performance status. Therefore, we suggest that adjuvant treatment be delivered earlier and completed for better outcomes in patients with resected PDAC.

## Supporting information

S1 Table Risk factors for disease-free survival. (DOCX)

S2 Table Reasons for discontinuing adjuvant treatment. (DOCX)

S1 Fig Distribution of adjuvant treatment timing. (TIF)

S2 Fig Kaplan-Meier curves of overall survival (A) and disease-free survival (B) in patients who received adjuvant treatment within 8 weeks, based on the median time of adjuvant treatment initiation (33 days). (TIFF)

S3 Fig Kaplan-Meier curves of overall survival (A) and disease-free survival (B), according to the adjuvant treatment strategy. (TIFF)
