# Supplementary material for: Early versus delayed initiation of adjuvant treatment for pancreatic cancer
Source: PLoS One. 2017 Mar 16;12(3):e0173960. doi: 10.1371/journal.pone.0173960 (PMC5354454; doi:10.1371/journal.pone.0173960)
Supplement: S1 Table — (DOCX) [file pone.0173960.s001.docx]

| **S1 Table. Risk factors for disease-free survival.** | | | | | | | | | | |  | |  | | |
| --- | --- | --- | --- | --- | --- | --- | --- | --- | --- | --- | --- | --- | --- | --- | --- |
|  |  | | **Univariate analysis** | | | | | | **Multivariate analysis** | | | | |  |  |
| **Risk factor** | **No.** | | | **HR (95%CI)** | | ***P* value** | | **HR (95%CI)** | | | | ***P* value** | | |  |
| Timing of the adjuvant treatment | |  | | |  | |  | | |  | |  | | |  |
| Early initiation | | 56 | | |  | |  | | |  | |  | | |  |
| Delayed initiation | | 57 | | | 1.649 (1.033–2.632) | | 0.036 | | | 2.029 (1.254–3.284) | | 0.004 | | |  |
| Age, years | |  | | |  | |  | | |  | |  | | |  |
| ≤65 | | 65 | | |  | |  | | |  | |  | | |  |
| >65 | | 48 | | | 0.861 (0.535–1.383) | | 0.535 | | |  | |  | | |  |
| Sex | |  | | |  | |  | | |  | |  | | |  |
| Female | | 40 | | |  | |  | | |  | |  | | |  |
| Male | | 73 | | | 1.239 (0.752–2.040) | | 0.400 | | |  | |  | | |  |
| CACI | |  | | |  | |  | | |  | |  | | |  |
| <6 | | 108 | | |  | |  | | |  | |  | | |  |
| ≥6 | | 5 | | | 2.078 (0.749–5.767) | | 0.160 | | |  | |  | | |  |
| Preoperative CA19-9 | |  | | |  | |  | | |  | |  | | |  |
| ≤100 U/mL | | 56 | | |  | |  | | |  | |  | | |  |
| >100 U/mL | | 57 | | | 1.574 (0.982–2.523) | | 0.059 | | |  | |  | | |  |
| Time of surgery | |  | | |  | |  | | |  | |  | | |  |
| 2006–2009 year | | 23 | | |  | |  | | |  | |  | | |  |
| 2010–2015 year | | 90 | | | 1.269 (0.737–2.185) | | 0.390 | | |  | |  | | |  |
| Surgical procedure | |  | | |  | |  | | |  | |  | | |  |
| Laparoscopic resection | | 24 | | |  | |  | | |  | |  | | |  |
| Open resection | | 89 | | | 1.952 (0.970–3.930) | | 0.061 | | |  | |  | | |  |
| Type of resection | |  | | |  | |  | | |  | |  | | |  |
| DP | | 36 | | |  | |  | | |  | |  | | |  |
| PD | | 77 | | | 0.901 (0.564–1.440) | | 0.664 | | |  | |  | | |  |
| Operating time | |  | | |  | |  | | |  | |  | | |  |
| ≤500 minutes | | 92 | | |  | |  | | |  | |  | | |  |
| >500 minutes | | 21 | | | 1.784 (1.021–3.118) | | 0.042 | | | 1.372 (0.759–2.480) | | 0.295 | | |  |
| Intraoperative transfusion | |  | | |  | |  | | |  | |  | | |  |
| No | | 93 | | |  | |  | | |  | |  | | |  |
| Yes | | 20 | | | 1.234 (0.702–2.169) | | 0.466 | | |  | |  | | |  |
| Longest diameter of primary tumor | |  | | |  | |  | | |  | |  | | |  |
| ≤2 cm | | 19 | | |  | |  | | |  | |  | | |  |
| >2 cm | | 94 | | | 2.327 (1.114–4.859) | | 0.025 | | | 1.709 (0.776–3.761) | | 0.183 | | |  |
| Nodal status | |  | | |  | |  | | |  | |  | | |  |
| Negative | | 43 | | |  | |  | | |  | |  | | |  |
| Positive | | 70 | | | 1.878 (1.139–3.095) | | 0.013 | | | 1.089 (0.639–1.856) | | 0.754 | | |  |
| Resection margin status | |  | | |  | |  | | |  | |  | | |  |
| **S1 Table. Risk factors for disease-free survival (continued).** | | | | | | | | | | | | | | |  |
| R0 | | 93 | | |  | |  | | |  | |  | | |  |
| R1 | | 20 | | | 0.980 (0.527–1.823) | | 0.949 | | |  | |  | | |  |
| Differentiation | |  | | |  | |  | | |  | |  | | |  |
| Well or moderate | | 99 | | |  | |  | | |  | |  | | |  |
| Poor | | 14 | | | 0.978 (0.501–1.912) | | 0.949 | | |  | |  | | |  |
| Angiolymphatic invasion | |  | | |  | |  | | |  | |  | | |  |
| Negative | | 55 | | |  | |  | | |  | |  | | |  |
| Positive | | 58 | | | 2.342 (1.448–3.788) | | 0.001 | | | 2.154 (1.267–3.662) | | 0.005 | | |  |
| Venous invasion | |  | | |  | |  | | |  | |  | | |  |
| Negative | | 66 | | |  | |  | | |  | |  | | |  |
| Positive | | 47 | | | 2.266 (1.418–3.620) | | 0.001 | | | 1.713 (1.033–2.838) | | 0.037 | | |  |
| Perineural invasion | |  | | |  | |  | | |  | |  | | |  |
| Negative | | 14 | | |  | |  | | |  | |  | | |  |
| Positive | | 99 | | | 2.909 (1.167–7.250) | | 0.022 | | | 1.830 (0.725–4.618) | | 0.201 | | |  |
| Postoperative complications | |  | | |  | |  | | |  | |  | | |  |
| No | | 67 | | |  | |  | | |  | |  | | |  |
| Grade I/II | | 30 | | | 0.952 (0.555–1.632) | | 0.858 | | |  | |  | | |  |
| Grade III/IV | | 16 | | | 0.962 (0.469–1.974) | | 0.917 | | |  | |  | | |  |
| Completed 6 cycles of treatment | |  | | |  | |  | | |  | |  | | |  |
| Yes | | 71 | | |  | |  | | |  | |  | | |  |
| No | | 42 | | | 3.333 (2.085–5.330) | | <0.001 | | | 2.991 (1.839–4.864) | | <0.001 | | |  |

HR, hazard ratio; CI, confidence interval; CACI, Charlson age-adjusted comorbidity index; CA19-9, carbohydrate antigen 19-9; DP, distal pancreatectomy; PD, pancreaticoduodenectomy; R0, macroscopically and microscopically negative resection margin; R1, microscopically positive resection margin.
