# Supplementary material for: Early versus delayed initiation of adjuvant treatment for pancreatic cancer
Source: PLoS One. 2017 Mar 16;12(3):e0173960. doi: 10.1371/journal.pone.0173960 (PMC5354454; doi:10.1371/journal.pone.0173960)
Supplement: S2 Table — (DOCX) [file pone.0173960.s002.docx]

| **S2 Table. Reasons for discontinuing adjuvant treatment.** | | |  |
| --- | --- | --- | --- |
|  | **Timing of the adjuvant treatment** | | |
| **Reasons for Discontinuation, no. (%)** | **Early group** | **Delayed group** | |
| No. of patients | 18 (32.1) | 24 (42.1) | |
| Recurrence | 11 (19.6) | 16 (28.1) | |
| Toxicity | 1 (1.8) | 0 (0) | |
| Patient decision | 5 (8.9) | 5 (8.8) | |
| Consultant decision | 1 (1.8) | 3 (5.2) | |
