# Supplementary figures and images for: Early versus delayed initiation of adjuvant treatment for pancreatic cancer
Source: PLoS One. 2017 Mar 16;12(3):e0173960. doi: 10.1371/journal.pone.0173960 (PMC5354454; doi:10.1371/journal.pone.0173960)

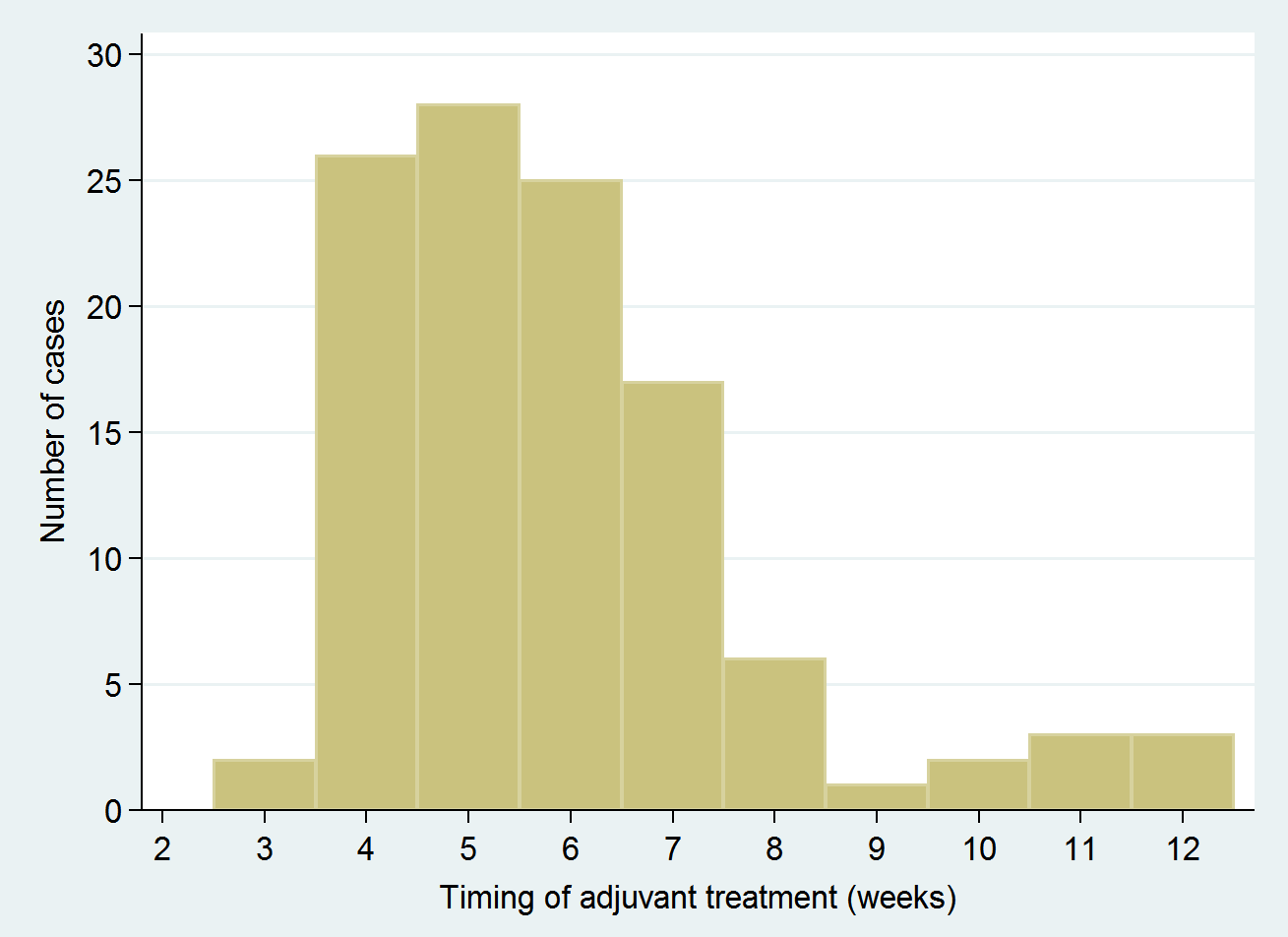

Supplement: S1 Fig — (TIF) [file pone.0173960.s003.tif]

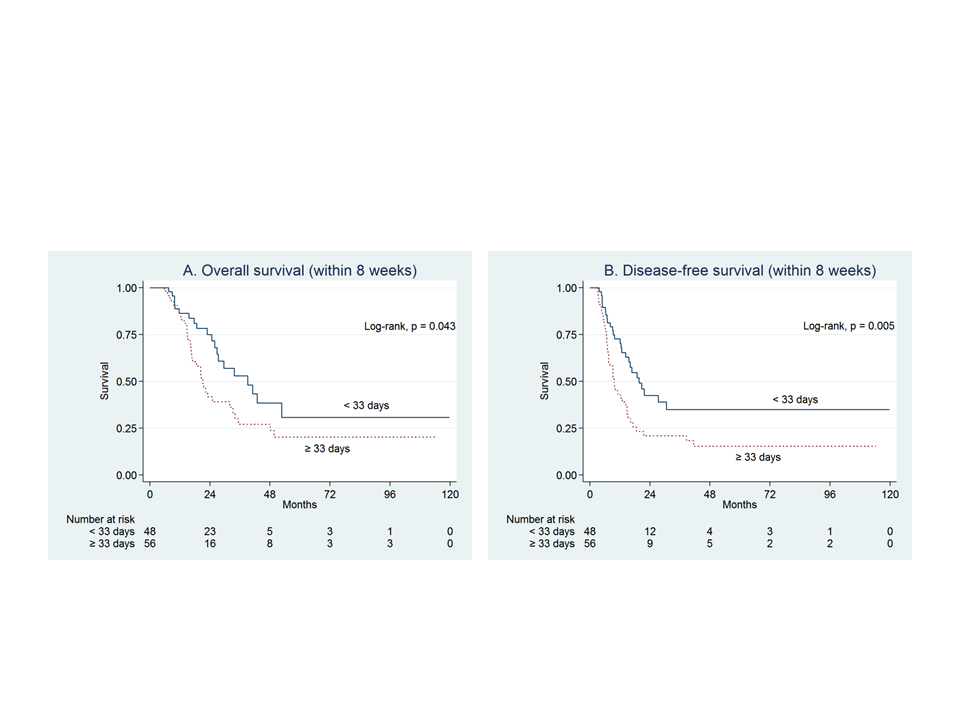

Supplement: S2 Fig — (TIFF) [file pone.0173960.s004.tiff]

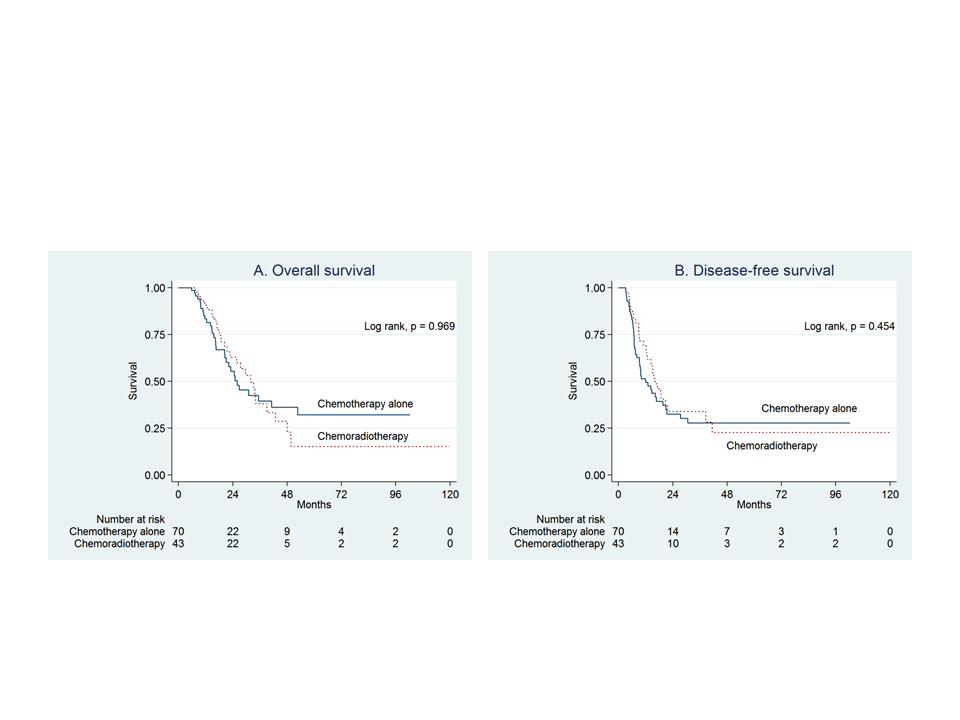

Supplement: S3 Fig — (TIFF) [file pone.0173960.s005.tiff]
